# Risk Factors and Interventions for Suicide in Huntington’s Disease—A Systematic Review

**DOI:** 10.3390/jcm13123437

**Published:** 2024-06-12

**Authors:** Alessandro Grimaldi, Isabella Veneziani, Laura Culicetto, Angelo Quartarone, Viviana Lo Buono

**Affiliations:** 1 Department of Nervous System and Behavioural Sciences, Psychology Section, University of Pavia, Piazza Botta, 11, 27100 Pavia, Italy; alessandro.grimaldi01@universitadipavia.it (A.G.); isabellamaria.veneziani01@universitadipavia.it (I.V.); 2 IRCCS Centro Neurolesi “Bonino-Pulejo”, S.S. 113 Via Palermo C. da Casazza, 98124 Messina, Italy; angelo.quartarone@irccsme.it (A.Q.); viviana.lobuono@irccsme.it (V.L.B.)

**Keywords:** Huntington’s disease, rare disease, suicide, neuropsychiatric symptoms, psychological assessment

## Abstract

**Background/Objectives**: Huntington’s disease (HD) is an autosomal dominant genetic disorder causing progressive neurodegeneration which, aside from symptomatic therapies for controlling psychological and motor problems, currently has no effective treatment. People who receive this diagnosis often feel disoriented and lost without guidance. Furthermore, HD patients are estimated to have a two to seven times greater risk of suicide death compared to the general population. The current review investigates the complex relationship between HD and suicide, seeking to identify key risk factors influencing suicidal ideation and behaviour in affected individuals. **Methods**: We conducted a systematic review following the PRISMA guidelines. Studies were searched for on the PubMed, Cochrane, and Web of Science databases, and 17 articles met the inclusion criteria. **Results**: The findings reveal that emotional strain, neuropsychiatric symptoms, and the absence of a cure contribute to heightened suicidal tendencies in HD patients. Critical periods for suicide risk coincide with early symptomatic stages of disease or the successive phase, with the loss of independence impacting on daily functioning. Risk factors associated with HD include a depressive mood, cognitive impairments, and a history of suicide attempts. **Conclusions**: From a prevention perspective, a comprehensive multidisciplinary and multidimensional approach could enhance the overall well-being of people with HD. In particular, screening for suicidal thoughts in people with HD could mitigate suicide risk.

## 1. Introduction

Huntington’s disease (HD), also known as Huntington’s chorea, is a genetic disorder, inherited in an autosomal dominant manner, that gradually affects the central nervous system [1,2,3]. HD is classified as a rare disease, and it affects a global population of around 3.92 per 100,000 individuals [4]. The incidence rate of this disease is slightly lower, standing at 0.47 cases per 100,000 person-years [4]. While there is some evidence that both the prevalence and incidence rates of HD are on the rise, it is possible that this increase may be attributed to better detection methods [4,5]. Notably, HD is more prevalent in Europe and North America as compared to Asia and Africa, possibly due to genetic factors [6,7,8,9]. The mutation responsible for this disease is found in the Huntingtin (HTT) gene, responsible for the production of the huntingtin protein that confers a toxic gain-of-function phenotype, resulting in neurodegeneration in the striatum [10,11]. Cortical pyramidal neurons that project to the striatum degenerate, and striatal neurons projecting to the substantia nigra also show a degeneration in presymptomatic patients [12,13,14]. In addition, HD patients experience a general reduction in brain volume, with different brain regions declining at varying rates [10,15].

HD is a complex condition that typically manifests in adulthood, most commonly between the ages of 30 and 50, although onset can occur at any age [16]. The disease is characterized by a range of symptoms, including chorea, which refers to uncontrollable and irregular movements of various parts of the body [17,18,19]. 

As the disease progresses, individuals with HD may experience a decline in cognitive functions, as memory, concentration, decision-making skills, and clarity of thought can all be impacted [20,21,22]. Additional symptoms may include anxiety, irritability, apathy, depression, and impulsive behaviour [23,24]. Neuropsychiatric symptoms and behavioural problems are significant aspects of the clinical spectrum of HD, impacting the quality of life for both patients and their families [25]. These symptoms are associated with the most distressing aspects of the illness and contribute significantly to the overall burden of the disease. HD gradually impairs an individual’s ability to perform daily activities, resulting in considerable disability [11]. Currently, there is no known cure for HD, and treatment focuses on managing the symptoms. 

Individuals with HD have a significantly higher risk of mortality by suicide compared to the general population [26]. Estimates suggest a two- to sevenfold increase in suicide prevalence within the HD population, positioning it as the third leading cause of death for HD patients following pneumonia and other infections [26,27,28]. This increased suicide risk in HD patients is due to a combination of factors. Neurological disorders like HD frequently manifest with neuropsychiatric symptoms (e.g., depression, anxiety, etc.) and cognitive decline (e.g., impulsivity and impaired problem-solving skills) [29]. The profound impact HD has on mental health can lead to feelings of hopelessness in some individuals. These co-occurring conditions can contribute to an increased likelihood of suicide. Furthermore, the progressive and currently incurable nature of HD can make patients feel trapped, potentially leading them to perceive suicide as an escape route [30]. Moreover, the two medications currently approved for the treatment of HD symptoms, tetrabenazine and deutetrabenazine, may also carry an increased risk of depression and suicide [31]. However, the exact association between tetrabenazine and suicide risk remains a topic of ongoing debate [32,33,34]. 

Suicidal behaviour encompasses a range of actions, spanning from attempts and preparatory behaviours to the ultimate act of suicide [35]. Suicidal ideation, on the other hand, involves the contemplation, consideration, or planning of suicide [36]. This systematic review focused on the studies that investigated the relationship between HD and suicide. This includes identifying and understanding the risk factors that may lead patients to consider this extreme option. Suicide is one of the few preventable causes of death in individuals with this neurodegenerative disorder. By better understanding these risk factors, targeted interventions can be developed to prevent suicide in this vulnerable population.

## 2. Materials and Methods

**Figure 1:**
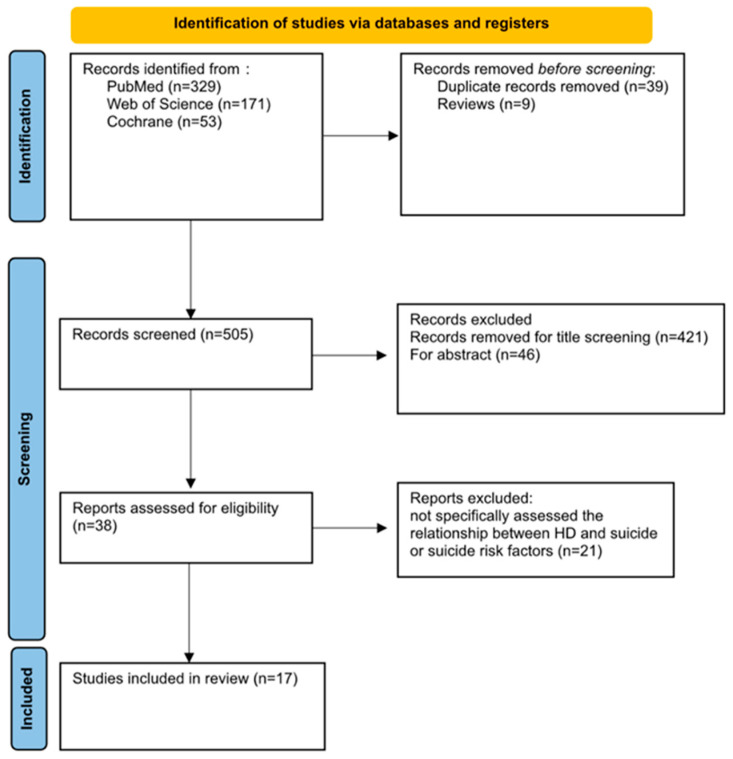
PRISMA flow diagram for research strategy.

This systematic review was conducted in accordance with the Preferred Reporting Items for Systematic Reviews and Meta-Analyses (PRISMA) guidelines [37] (Figure 1). This review has been registered on OSF (n) 10.17605/OSF.IO/AEUTP.

### 2.1. Search Strategy 

Articles were selected from research databases, namely PubMed, Cochrane, and Web of Science (Table 1), using the following search terms: (“suicid”[All Fields] OR “suicidal ideation”[MeSH Terms] OR (“suicidal”[All Fields] AND “ideation”[All Fields]) OR “suicidal ideation”[All Fields] OR “suicidality”[All Fields] OR “suicidal”[All Fields] OR “suicidally”[All Fields] OR “suicidals”[All Fields] OR “suicide”[MeSH Terms] OR “suicide”[All Fields] OR “suicides”[All Fields] OR “suicide s”[All Fields] OR “suicided”[All Fields] OR “suiciders”[All Fields]) AND (“huntington”[All Fields] OR “huntington s”[All Fields] OR “huntingtons”[All Fields]).

All articles were reviewed based on their titles, abstracts, and full texts by two investigators (AG and IV) who independently performed data collection to reduce the risk of bias (i.e., the bias of missing results, publication bias, time lag bias, and language bias). These researchers read the full-text articles deemed suitable for the study, and in case of disagreement on the inclusion and exclusion criteria, the final decision was made by a third researcher (LC). The list of articles was then refined for relevance, revised, and summarized, with the key topics identified from the summary based on the inclusion/exclusion criteria. 

The inclusion criteria were as follows: (i) articles that enrolled adult patients with HD; (ii) studies that specifically assessed the relationship between HD and suicide or suicide risk factors; (iii) articles published in the English language only.

We excluded the following types of studies: (i) case studies and reviews; (ii) articles with a lack of an assessment of suicide risk; (iii) duplicate studies; (iv) animal studies; (v) non-English studies.

### 2.2. PICO Model 

We employed the PICO (Population, Intervention, Comparison, and Outcome) model to shape our research question [38]. 

Population

Adult individuals diagnosed with HD.

Intervention

This systematic review examines the relationship between HD and suicide, focusing on identifying risk factors associated with suicidal ideation and behaviour within the HD population. Additionally, it explores potential interventions to mitigate suicide risk in individuals with HD.

Comparison

The review compares different studies in the literature to identify common risk factors for suicide in HD patients, including a depressive mood, neuropsychiatric symptoms, cognitive impairments, and challenging transition periods during the progression of the disease. 

Outcome

The primary outcomes of interest include understanding the prevalence of suicidal ideation, behaviour, and suicide among individuals with HD, as well as identifying effective interventions for preventing suicide within this population. Additionally, the review aims to highlight gaps in current understanding and areas for future research to improve clinical approaches and patient outcomes in HD management.

### 2.3. Study Selection 

To guarantee the impartiality of the study selection process, two authors (IV and AG) independently extracted data, resolving any discrepancies through collaborative discussion and consultation with a third author (LC). Each article was assessed by at least three authors independently, and if there were any disagreements, the other authors were consulted. The required data were obtained from the full-text articles. If any crucial information was absent from the original studies, their authors were contacted. This approach was implemented to eliminate any potential bias and to reinforce the study findings’ validity and reliability.

### 2.4. Data Extraction and Analysis

After selecting the full-text, the studies were analysed, and data were extracted. The extracted data were tabulated using Microsoft Excel (Version 2021). To evaluate the agreement between the two reviewers (I.V. and A.G.), the kappa statistic was used. The kappa score, with an accepted threshold for substantial agreement set at >0.61, was interpreted to reflect excellent concordance between the reviewers. The criterion ensures a robust evaluation of the inter-rater reliability, emphasizing the achievement of a substantial level of agreement in the data extraction process.

### 2.5. Risk of Bias within Individual Studies 

IV, AG, and VLB independently assessed the risk of bias for each study, which was cross-checked by LC. The risk of bias in was assessed using the Risk of Bias in Non-Randomized Studies of Exposure (ROBINS-E) (2023) tool [39], which comprises seven domains: (i) bias due to confounding; (ii) bias arising from the measurement of exposure; (iii) bias in the selection of participants for the study (or in the analysis); (iv) bias due to post-exposure interventions; (v) bias due to missing data; (vi) bias arising from the measurement of the outcome; and (vii) bias in the selection of the reported results.

## 3. Results

A total of 553 articles were identified through searches of the PubMed, Cochrane, and Web of Science databases. Of the articles identified, 39 duplicate articles and 9 reviews were deleted; 421 studies were excluded after title screening, and 46 were excluded after abstract screening; 21 studies underwent full-article screening to assess eligibility. Finally, only 17 articles met the inclusion criteria (Figure 1).

### 3.1. Synthesis of Evidence

This systematic review investigates the concerning phenomenon of suicide within the HD population. The studies included primarily address the elevated risk of suicide in HD patients and the potential interventions that could be used to mitigate this phenomenon, highlighting the role of physicians in navigating end-of-life decisions (Table 2).

The results suggest a potential link between the high prevalence of suicidal ideation, suicidal behaviour, and completed suicides in HD patients and the emotional strain associated with a progressive, incurable disease [40]. The co-occurrence of prevalent psychopathology in HD may further contribute to this increased risk. Three longitudinal studies demonstrate a clear association between a depressive mood and suicidal ideation in HD patients across both premotor and motor symptomatic stages [41,42,43]. Notably, a depressive mood has been identified as a predictive factor for both suicide attempts and completed suicides during the prodromal stage of the disease [42]. Several other factors linked to an increased risk of suicide include anxiety, aggression, a history of suicide attempts, and the use of benzodiazepines [43]. Neuropsychiatric symptoms can manifest at any stage of HD [25] and often precede the onset of motor symptoms. The combined effect of neuropsychiatric symptoms, including depression, irritability, apathy, and obsessive compulsive behaviours with cognitive impairments such as impulsivity, impaired abstract reasoning, and problem-solving deficits, can significantly increase the risk of suicide [44]. Nonetheless, a consistent association between suicide and mental illness was not consistently observed among patients with HD. Instead, suicide appeared to be perceived as a rational response to the harsh realities of living with HD, such as the progressive physical decline and the demanding nature of long-term intensive care [45]. The prospect of enduring a debilitating and ultimately fatal disease with no effective treatment options can induce feelings of helplessness and entrapment, prompting some individuals to consider suicide as a means of escape [30].

There are two critical periods during which individuals with HD are at the highest risk of committing suicide [46]: (1) the initial stage, when early symptoms begin to manifest, and (2) the later stage, when patients become heavily dependent on others for daily functioning. This transition can be particularly psychologically challenging [41,47,48]. Research suggests that individuals with a genetic predisposition to HD who start exhibiting initial symptoms may be particularly vulnerable to suicide risk [43,46]. Furthermore, several studies indicate that suicide is more likely to occur in the early stages of HD. This increased risk may be due to a heightened awareness of the disease’s progression and the ability to plan and carry out a suicide attempt [49,50].

Our literature review also revealed that suicidal ideation plays an important role in the act of suicide [36]. Despite its acknowledged presence within the HD population, the psychological impacts of predictive testing for HD have not been thoroughly investigated in relation to suicidal thoughts [51]. A study investigating the psychological repercussions of predictive testing for HD revealed that a significant portion of participants (50%) had experienced or were currently experiencing suicidal thoughts [52]. Additionally, the prevalence of these thoughts tended to increase over time, suggesting that they may serve as a consistent indicator of psychological distress and hopelessness [53,54]. There is also a potential link between the use of antidepressant or anxiolytic medications in HD patients and the onset of suicidal ideation or suicide attempts [30,43]. 

The abuse of substances, including alcohol and drugs, is a known risk factor for suicide in the general population and has been linked to HD as well. This association can contribute to disorientation, impulsivity, and risky behaviour, further increasing suicide risk in HD patients [41,46,53].

A history of suicide attempts is a particularly critical factor, serving as a significant warning signal. Individuals with HD who have previously attempted suicide are at a heightened risk of subsequent attempts, potentially indicating profound distress and despair [45]. Addressing suicidal ideation is essential in HD management, demanding effective intervention strategies. A related and ethically complex issue is the role of euthanasia and physician-assisted suicide (PAS) by patient request or through advance directives [54]. Exploring these aspects necessitates a discussion about the professional role of a physician in relation to a patient’s autonomy in end-of-life decisions [55].

**Table 2:** Characteristics of the studies included.

Study	Design	Aims	Patients	Measures	Major Findings
Farrer LA (1986) [51]	Retrospective Study	Explore the implications for preclinical testing of individuals at risk of HD based on the incidence of suicide and attempted suicide	A total population of 831 HD patients	AQ	The study revealed that 5.7% of fatalities among individuals affected by the condition were due to suicide, and 27.6% of patients made at least one suicide attempt.
Di Maio L et al. (1993) [49]	Cross-sectional Study	Assess the risk of suicide in HD	2793 individuals	Family history and affected questionnaire	Suicide was identified as the reported cause of death in 205 individuals, accounting for 7.3% of the studied subjects.
Robins Wahlin TB et al. (2000) [50]	Cross-sectional Study	Assess the possible impacts of presymptomatic testing	600 patients with HD and 3000 individuals at risk	GHQ-30; LSA; AQ; SIBS; LSI	Both groups showed increased SI. Non-carriers had more suicide attempts, but both groups had significant psychiatric dysfunction.
Paulsen JS et al. (2005) [46]	Prospective Longitudinal Study	To identify critical periods of suicide risk in HD	2835 subjects who received a diagnosis of HD	UHDRS	Suicide is more likely to occur in the early stages of HD and when patients experience the loss of autonomy.
Larsson MU et al. (2006) [52]	Prospective Follow-up Study	Reports a two-year follow-up of psychological effects of predictive testing for Huntington’s disease	35 carriers and 58 non-carriers of the HD gene.	UHDRS; GHQ-30; BDI; SIBS; LSI; LSA	Before predictive testing, both carriers and non-carriers showed elevated SI. Carriers experienced rising depression scores and more frequent suicidal thoughts over time.
Fiedorowicz JG et al. (2011) [42]	Prospective Study	Determine risk factors for suicidal behaviour, defined as suicide or attempted suicide, in prodromal HD	735 cases with HD gene expansion and 194 non-gene-expanded controls.	PSS; LES; and UHDRS	A history of suicide attempts and the existence of depression were associated with suicidal behaviour in the prodromal stage of HD.
Wetzel HH et al. (2011) [30]	Cross-sectional Study	Improved understanding of risk factors for suicide in HD	4000 patients with or at risk of HD	UHDRS	Psychiatric symptoms were linked to elevated rates of SI in HD.
Hubers AA et al. (2012) [41]	Cross-sectional Study	Study the prevalence and traits of suicidality, encompassing both thoughts and actions, in individuals with HD	152 mutation carriers and 56 non-carriers	PBA	A connection has been established between a depressive mood and the potential prediction of suicidality in HD.
Halpin M (2012) [45]	Qualitative Study	Evaluate if suicide is attributed to mental pathology or the concept of rational suicide	20 individuals with HD and 10 informal caregivers	Semi-structured interview	Study participants did not link suicide with mental pathology. Instead, they perceived suicide as a response to the reality of living with HD.
Hubers AA et al. (2013) [43]	Cross-sectional Study	Investigate correlates and predictors of SI in HD	2106 HD mutation carriers	PBA-s; HDQLIFE	Depressed mood and benzodiazepine use predicted new-onset SI, while prior suicide attempts did not.
Booij SJ et al. (2014) [54]	Cross-sectional Study	Examine the occurrence of suicidal thoughts in individuals diagnosed with HD or identified gene carriers	134 patients	TFC subscale of the UHDRS; MMSE	From a total of 101 respondents, 75% admitted to end-of-life thoughts, and 11% considered care, while 64% contemplated euthanasia or PAS.
Anderson KE et al. (2016) [55]	Cross-sectional Study	Investigate correlations between, mood disorders, CAG expansion status, and motor symptoms with SI in at-risk HD patients	801 subjects	UHDRS, Barratt Impulsivity Scale	Individuals with suicidal ideation showed higher levels of behavioural symptoms, with feelings of hopelessness and anxiety being strongly associated with this ideation.
van Duijn E et al. (2018) [40]	Cross-sectional Study	Examine the complete range of suicidality, encompassing SI, suicidal actions, and self-injurious behaviour	1451 HD gene expansion carriers	C-SSRS, UHDRS, PBA	Among HD gene expansion carriers, 6.5% reported a lifetime suicide attempt. SI was associated with a depressed mood, with irritability playing a lesser role.
Wesson M et al. (2018) [48]	Cross-sectional Study	Examine SI and the impact of assessment modality in HD	496 participants with premanifest or manifest HD	PBA-s; 16	Individuals with HD were more likely to endorse suicidal ideation via self-report than via in-person interview
van Duijn E et al. (2021) [44]	Prospective Global Study	Investigate the incidence of completed suicide and suicide attempts in HD	20,912 participants, comprising 15,924 HDGECs and 4988 non-HDGECs	C-SSRS; UHDRS	Suicide rate in HDGEC: 72 per 100,000 person-years, compared to 8 per 100,000 person-years in non-HDGEC settings. Proportionate mortality from suicide in HDGECs: 4.6%.
Alothman D et al. (2022) [56]	Retrospective Study	Examine the relative risk of suicide mortality in HD	594,674 individuals	CPRD; HES; ONS	Risk of death from suicide was markedly elevated in younger individuals with HD compared to HC people.
Rocha NP et al. (2022) [47]	Observational Study	Pinpoint clinical factors linked with a past occurrence of depression and suicidal tendencies in individuals carrying the HD gene	Periodic data set 4; N = 11,582	Binary logistic regression	The prevalence of depression and suicidality were high among HD gene carriers.

Legend: HD = Huntington’s disease; HDGEC = High-Dependency Geriatric Care; HDGECs = Huntington’s disease gene expansion carriers; UHDRS = Unified Huntington’s Disease Ratings Scale; PBA = Problem Behaviours Assessment; PBA-s = Problem Behaviours Assessment—Short; SI = suicidal ideation; C-SSRS = Columbia Suicide Severity Rating Scale; TFC = total functioning capacity; MMSE = mini mental state examination; PAS = physician-assisted suicide; GHQ-30 = General Health Questionnaire-30; LSA = The Life-Styles Assessment; AQ = attitude questionnaire; SIBS = Survey of Innovation and Business Strategy; LSI = Life Styles Inventory; BDI = Beck Depression Inventory; SIBS = The Self-Injurious Behaviour Scale; LSI = life Satisfaction index; LES = Life Experiences Survey; PSS = Psychiatric Status Schedule; HCs = healthy controls; CPRD = Clinical Practice Research Datalink; HES = Hospital Episode Statistics; ONS = Office for National Statistics; HDQLIFE = Huntington’s disease quality life questionnaire.

### 3.2. Risk of Bias

**Figure 2:**
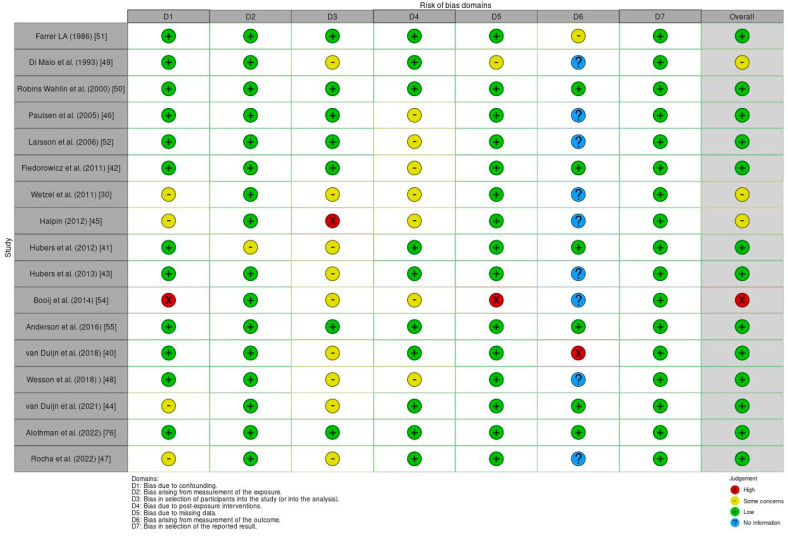
Risk of bias (ROBINS-E) of the studies included.

The ROBINS-E tool [39] was used to assess the risk of bias of the articles included in this review. Figure 2 shows a summary of the risk of bias assessment, while the graphs depict the distribution of bias concerns across the included studies. Out of all the studies assessed, one study [54] showed a high overall risk of bias due to confounding and missing data. One study [45] reported a high risk of bias in the selection of participants for the study (or in the analysis), and another one [40] had a high risk of bias from the measurement of the outcome. Additionally, four studies [30,44,45,47] displayed some concerns about bias due to confounding. Further, the studies conducted by Hubers et al. (2012) [41] and Di Maio et al. (1993) [49], respectively, exhibited some concerns about bias arising from measurement of the exposure and from missing data. Moreover, some concerns about bias in the selection of participants for the study (or in the analysis) were found in different studies [30,40,41,43,44,47,48,49,54]. Finally, some studies [30,42,45,46,48,52,54] showed some concerns about bias due to post-exposure interventions, and one [51] showed some concerns about bias from measurement of the outcome.

## 4. Discussion

Suicide is a leading cause of death in individuals with HD [26], and it stands out as one of the few preventable causes of premature mortality associated with this disorder. The incidence of suicidal ideation, suicidal behaviour, and completed suicide are notably higher in patients coping with incurable diseases, potentially due to the immense emotional strain and specific psychopathological factors associated with their condition [57]. Nonetheless, suicide prevention efforts are paramount, with the World Health Organization aiming to decrease the global suicide rate by one third by 2030 [58]. In the context of HD care, integrating psychological services into treatment models can play a crucial role in achieving this goal. These integrated care models help alleviate the psychological burden associated with HD, not only improving patients’ well-being but also reducing healthcare costs [59,60]. The studies examined suggested that depression, anxiety, and neuropsychiatric symptoms significantly contribute to the risk of suicide in HD and require considerable attention from a clinical point of view [42]. Further, personality traits such as hopelessness, neuroticism, psychoticism, perfectionism, aggression, and irritability are associated with the highest risk of suicidality [30]. The alteration of frontal–subcortical circuitry in HD has been suggested as a possible cause of difficulties in inhibiting emotionally driven behaviour, potentially leading to impulsivity and suicidal acts [61]. Because of its autosomal dominant nature, individuals with HD often witness the progression of the disease in family members, fostering a belief that they too will experience a similar fate, especially in the absence of effective treatments [62]. Effective HD treatment should not only focus on managing symptoms but also aim to improve the overall well-being of those affected. Patients are concerned with more than just their test results or symptom severity; they are deeply impacted by how HD affects their daily lives [60]. Recognizing the importance of mental health is crucial in shaping the outcomes of HD.

The topic of suicide is also linked to important ethical considerations and the responsibility of clinicians. Many individuals with HD tend to predominantly express their end-of-life wishes through euthanasia or PAS [54]. Since euthanasia and PAS are legal in different countries, the consideration of these aspects leads to a discussion about the professional role of a physician in relation to the personal autonomy of a patient [63]. 

Recently, there has been a significant increase in efforts to develop psychological support mechanisms for individuals with HD, particularly aiming to integrate a psychologically informed clinical framework to better understand and address distress within this population [64,65]. Consequently, several psychological interventions have been adapted and tested for HD, showing promising initial results [66,67,68,69]. However, the overall evidence regarding the effectiveness of these interventions in HD patients remains limited. A recent literature review [70], supported by guidelines from the British Psychological Society [71], highlighted a concerning lack of data and a scarcity of dedicated psychological services for HD patients. Depression, one common psychological challenge for HD patients, has received limited attention in terms of psychological interventions. Although CBT-informed interventions have shown promising effects in reducing low mood [67,72], robust evidence is lacking, necessitating more comprehensive investigations. Psychologists should be integral members of HD care teams globally, yet this is not consistently the case. Italy, in particular, exhibits deficiencies in this regard, with only one recent study [60] addressing psychological interventions for HD and the current availability and distribution of mental health services for HD within communities remaining uncertain. This highlights the urgency of improving clinicians’ understanding of HD and its psychological implications, promoting collaboration with patient organizations, and adopting a holistic approach to psychological care that addresses the diverse needs of individuals and families affected by HD. To enhance accessibility, personalized interventions, such as online therapy and home visits, should be considered. Additionally, in future, systemic approaches that involve the entire family system should be prioritized in the public provision of psychological services for HD in Italy [60].

Despite the high suicide risk among individuals with HD, there is a significant lack of evidence regarding effective interventions for managing suicidal ideation and suicide attempts. Early intervention is crucial in preventing suicide in this population. Suicide prevention can be approached in three main ways: preventing suicidal behaviour before it starts, intervening with people at risk, and supporting those affected by suicide. The first approach, primary prevention, aims to reduce the overall incidence of suicide in the community [73]. This might involve promoting mental health awareness or addressing social factors that can contribute to suicidal feelings. The second approach, secondary prevention, focuses on individuals already experiencing suicidal thoughts [73]. This involves identifying high-risk individuals and providing them with necessary resources and support to help them cope. The third approach, tertiary prevention, comes after a suicide attempt or completion [73,74]. It aims to prevent further suicides, especially those that might be influenced by the first incident. Secondary prevention is a crucial area, but it often does not receive the focus it deserves, partly due to the ongoing translation of research into practical interventions. For individuals experiencing suicidal ideation, specific therapies addressing the recurrence of suicidal thoughts and behaviours, ensuring treatment adherence, and tackling other factors commonly associated with suicidality can be highly beneficial [75]. Therefore, it is advisable to routinely assess suicide risk among patients diagnosed with HD in both primary and secondary care settings, paying particular attention to younger patients [56]. 

### 4.1. Future Directions

Individuals diagnosed with HD often feel anxious about the uncertain future and what it holds for them since there is currently no cure for the disease. However, the field of HD therapies is rapidly advancing, offering hope for the future. Current therapies for HD mainly focus on managing symptoms through a multidisciplinary approach encompassing both pharmacological and non-pharmacological treatments [76]. One of the main limitations of the current therapies is their inability to specifically target the root mechanisms of HD, leading to the persistent propagation of pathological processes that hinder treatment efficacy [77]. To address these limitations, future research is shifting focus towards more targeted approaches such as neurotransmission and mitochondrial function, as well as RNA- and DNA-targeted therapies [78]. Similarly, it is important for future studies to prioritize addressing the issue of suicide in this disease rather than treating it as a secondary concern. Given the potentially preventable nature of suicide in HD, there is a critical need for the development and assessment of targeted interventions designed to mitigate suicidal ideation and behaviour in this population. Such interventions should encompass a multifaceted approach, potentially including psychotherapeutic modalities, pharmacological treatments, and robust psychosocial support programs [30]. Furthermore, the integration of standardized suicide risk assessment and management protocols into routine HD clinical care is paramount. This comprehensive strategy holds the potential to significantly reduce suicide-related mortality and improve the overall well-being of individuals living with HD. However, there is still a lot of work to be conducted before these treatments can be made available to HD patients. Nonetheless, recent developments are promising and could mark the beginning of a new era in HD therapies.

### 4.2. Limitations

This systematic review investigates the complex relationship between HD and suicide, seeking to identify key risk factors influencing suicidal ideation and behaviour in affected individuals. The multifaceted nature of the interplay between factors in HD necessitates a comprehensive and multidimensional approach.

Overall, 17 studies were chosen through a comprehensive search strategy used across various databases. To ensure transparency and eliminate bias, clear inclusion and exclusion criteria were established. The review acknowledges that limitations exist due to the absence of standardized protocols, and a meta-analysis could not be conducted, as the research on suicide tends to lean more towards qualitative research. The methodologies employed across the studies varied considerably. These factors, combined with the limited number of studies, indicate that further research is necessary to improve the applicability of these findings.

## 5. Conclusions

Suicide presents a significant and preventable risk among individuals with HD, underscoring the need for targeted interventions and support mechanisms. Factors like depression, neuropsychiatric symptoms, and loss of autonomy contribute to increased suicidal ideation, highlighting the importance of routine screening and early intervention [29,44]. Although the lack of standardized protocols and varying definitions pose challenges, studies emphasize the necessity of addressing psychiatric symptoms in clinical care. Despite the complex nature of HD, regular assessment of suicide risk is crucial. Future research should focus on identifying protective factors and addressing gaps in understanding the heightened suicide risk in HD patients, aiming to improve clinical approaches and patient outcomes.

## Figures and Tables

**Table 1:** Search strategy.

Databases	PubMed, Cochrane, and Web of Science
Search Terms	(“suicid”[All Fields] OR “suicidal ideation”[MeSH Terms] OR (“suicidal”[All Fields] AND “ideation”[All Fields]) OR “suicidal ideation”[All Fields] OR “suicidality”[All Fields] OR “suicidal”[All Fields] OR “suicidally”[All Fields] OR “suicidals”[All Fields] OR “sui-cide”[MeSH Terms] OR “suicide”[All Fields] OR “suicides”[All Fields] OR “suicide s”[All Fields] OR “suicided”[All Fields] OR “suiciders”[All Fields]) AND (“huntington”[All Fields] OR “huntington s”[All Fields] OR “huntingtons”[All Fields])
Exclusion Criteria	(i) case studies and reviews; (ii) articles with a lack of an assessment of suicide risk; (iii) duplicate studies; (iv) animal studies; (v) non-English studies.
Inclusion Criteria	(i) articles that enrolled adult patients with HD; (ii) studies that specifically assessed the relationship between HD and suicide or suicide risk factors; (iii) articles published in the English language only.
Review Process	Titles, abstracts, and full texts were reviewed by two investigators (AG and IV) independently. A third researcher (LC) made the final decision in case of disagreement on inclusion/exclusion criteria.

## Data Availability

Data are contained within the article.
